# Analysis of the effects of stent-induced deformation on the hemodynamics of MCA aneurysms

**DOI:** 10.1038/s41598-023-39685-3

**Published:** 2023-07-31

**Authors:** Conggang Huang, Xueqin Zhou, Faliang Duan, Ruixue Li, Ming Luo, Zhihua Luo, SValiallah Mousavi

**Affiliations:** 1 Neurosurgery Department, Wuhan NO.1 Hospital, Wuhan, 430022 Hubei China; 2 Intensive Care Unit, Wuhan Sixth Hospital, Wuhan, 430014 Hubei China; 3 Department of Mechanical Engineering, Technical and Vocational University (TVU), Tehran, Iran

**Keywords:** Biomedical engineering, Mechanical engineering

## Abstract

The use of a stent to coil an aneurysm can alter the position of the main blood vessel and affect blood flow within the sac. This study thoroughly examines the impact of stent-induced changes on the risk of MCA aneurysm rupture. The research aims to assess the effects of coiling and vessel deformation on blood flow dynamics by comparing the OSI, WSS, and blood structure of two distinct MCA aneurysms to identify high-risk areas for hemorrhage. Computational fluid dynamics is used to model blood flow. The results indicate that aneurysm deformation does not always decrease the risk of rupture, and coiling is more effective in occluding blood flow than aneurysm deformation.

## Introduction

In the United States, the rupture of intracranial aneurysms is a significant challenge, as 30,000 cases of ruptured aneurysms are reported in US patients each year. The rupture of an aneurysm can lead to death or debilitation, and endovascular coils are the primary conventional treatment technique used to obstruct aneurysms and reduce the risk of rupture. Previous reports suggest that the quality of coiling plays a crucial role in reducing the risk of aneurysm rupture, particularly in the aneurysm ostium region, which experiences high-velocity impinging blood flow due to poor filling^1,2^. If there is a residual neck or unoccupied neck remnant, growth, recanalization, and rupture may occur, making treatment essential^3–5^.

The usage of shape polymer foam is suggested as one of the reliable methods for occlusion of the aneurysm^6,7^. This technique substantially decreases the blood flow into the sac and makes a framework for thrombus establishment^8,9^. The application of the stent is helpful for the efficient coiling of the cerebral aneurysm^10,11^. The usage of the polymer foam as coils results in optimistic long-standing healing based on the in vivo studies. On the other side, the filling of the aneurysm could effectively have been done by the usage of a stent since this permits the coiling of the aneurysm with higher density^12–14^. In addition, the usage of the stent allows higher coiling near the neck area which is susceptible to rupture by the reason of the high flow rate.

The side effect of stent usage is the deformation of the parent vessel which also has a great impact on the performance of the occlusion of blood stream into the main sac area^15–18^. The recognition of the blood hemodynamic under impacts of the stent-induced deformation offers valuable information for surgeons for estimation of long-term treatment of the aneurysm^19–22^. The post-interventional impacts of stents have been investigated in limited research and results considerably vary based on the types and shapes of aneurysms^23–27^.

In this study, the hemodynamic analyses of the MCA aneurysms have been performed to reveal the impacts of the stent on the rupture risk of the saccular aneurysm after the deformation of the parent vessel. To study the hemodynamic impacts of the aneurysms, computational fluid dynamics is applied for the analysis of the bloodstream inside the MCA aneurysm at two stages of deformations. In addition, the influence of coiling porosity is also investigated after the deformation of the parent vessel.

## Governing method and applied methodology

It is confirming that all methods were carried out in accordance with relevant guidelines and regulations. Besides, all experimental protocols were approved by of the Ca’ Granda Niguarda Hospital and it is confirmed that informed consent was obtained from all subjects and/or their legal guardian(s). All study are approved by Ca’ Granda Niguarda Hospital ethics committee^28^.

The simulation of blood hemodynamics inside the MCA aneurysm with/without deformation by stent is done via computational fluid dynamics^29–32^. The transient form of Navier–stokes is used for the modeling of blood flow through the vessel and sac section^33–35^. The one-way FSI model is used to apply the impacts of the vessel interaction under the impacts of blood pressure^36–39^. This technique is extensively used for the simulation of deformable domain where fluid has impacts on the solid wall^40–42^. Computational technique has been used in different applications of engineering^43,44^ and medical devices^45,46^. In this approach, the pressure force on the vessel would result in the defamation of the sac and vessel. The bloodstream is assumed transient with cardiac cycle and non-Newtonian^28^. Casson model is applied for the calculation of the blood viscosity^29^.

**Figure 1:**
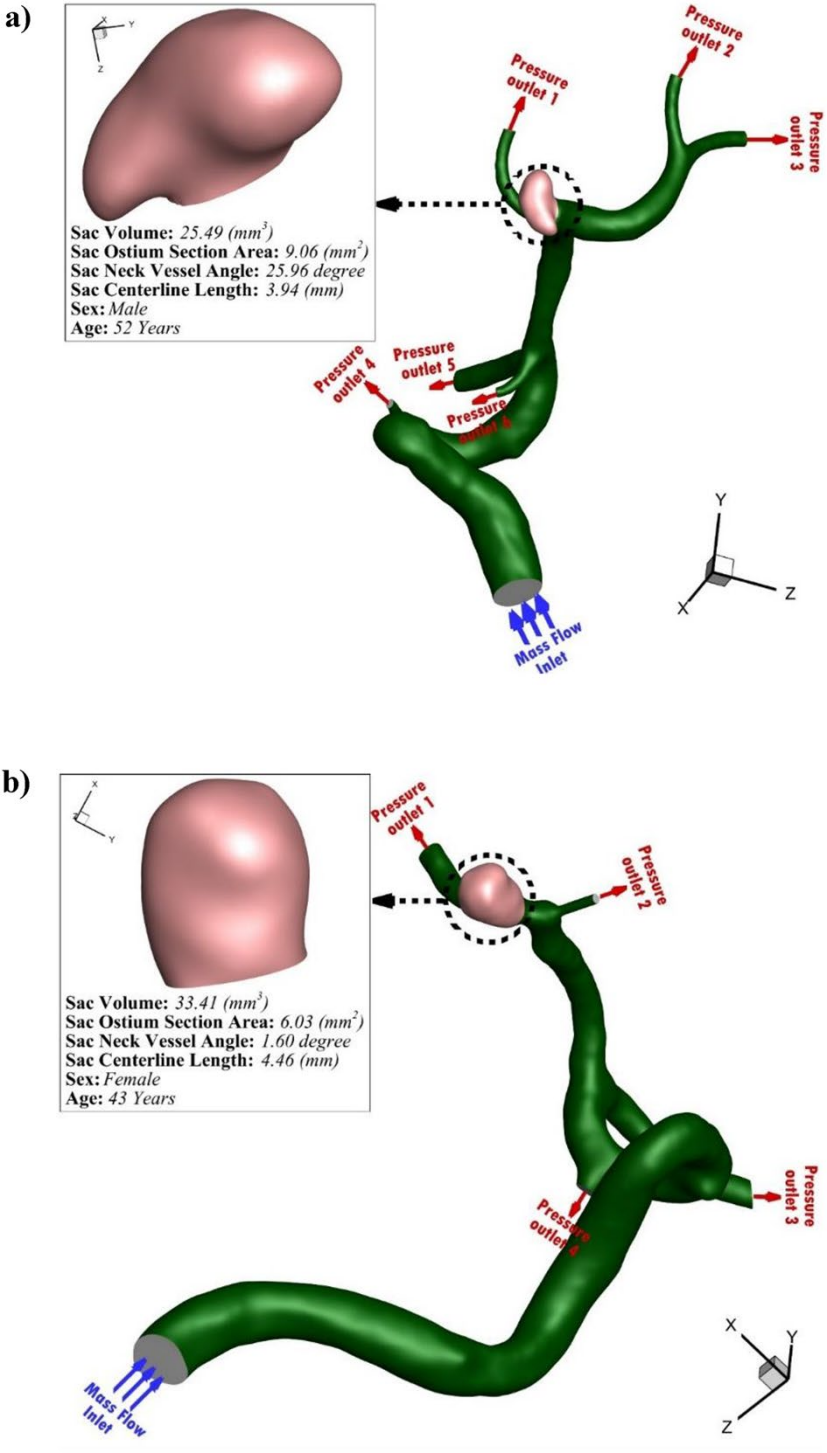
Selected MCA aneurysms.

**Figure 2:**
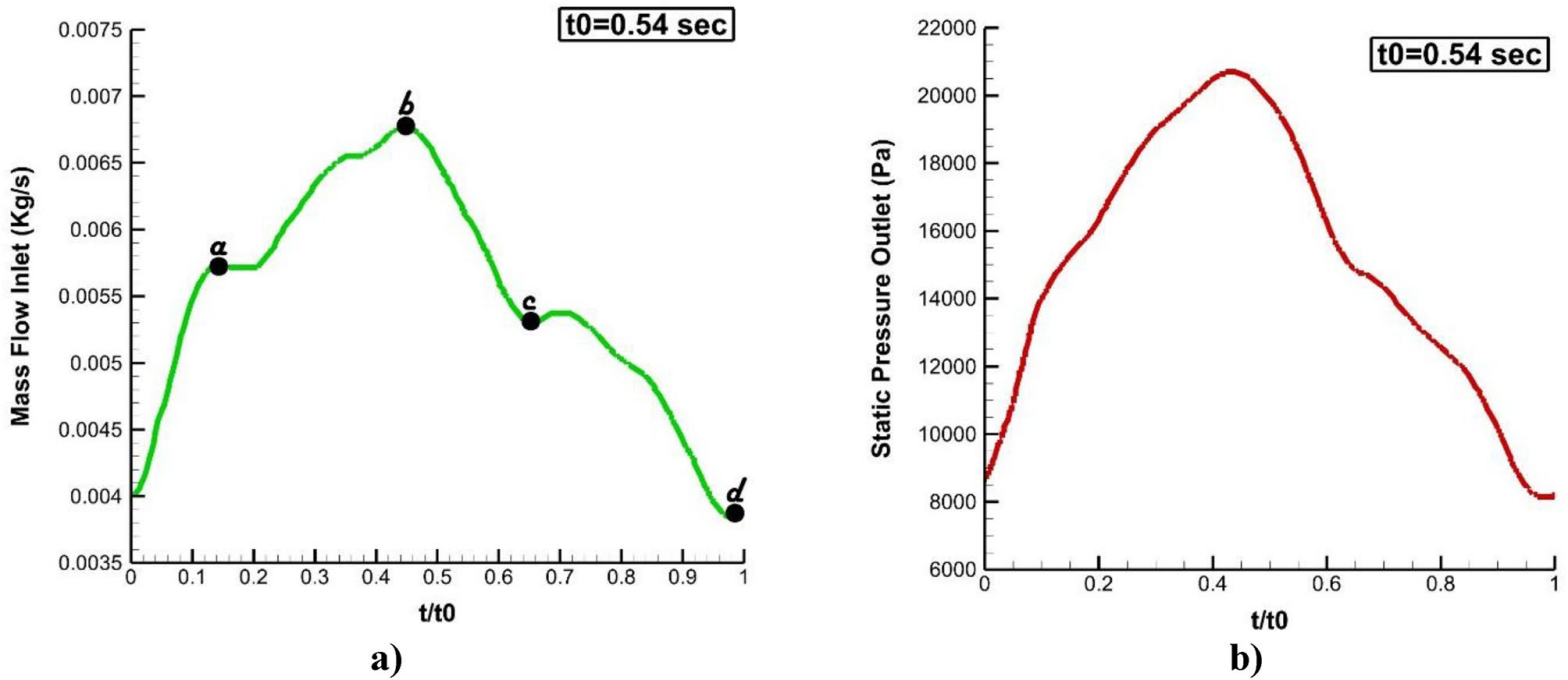
Applied boundary condition (**a**) inlet, (**b**) outlet (t_0_ = 0.54 s).

The geometries of the selected MCA aneurysms are displayed in Fig. 1. The details of the aneurysm characteristics (i.e. sac volume, sac ostium section area, sac neck vessel angle, and …) are also presented in Fig. 1. The geometries of the chosen MCA aneurysms are obtained from aneurisk website which is related to Emory University^28^. This figure also demonstrates the applied boundary condition for the chosen aneurysms. Inflow blood is applied by mass flow rate pattern (Fig. 2a) while outflow is a pressure outlet with a specific profile (Fig. 2b). This study reports the data associated with the 3rd cardiac cycle. The OSI index is calculated at the end of the third cardiac cycle. The hemodynamic results of the four stages (specified in Fig. 2) are presented and compared. Table 1 presents the mass flow rate of these stages. Since the maximum blood flow rate happens at stage b (peak systolic), the contour of pressure and WSS of this stage are presented^29^.

**Table 1:** Mass flow rate of blood stream in different stages.

	t (s)	m (kg/s)
a	0.06	0.0057
b	0.24	0.0068
c	0.36	0.0053
d	0.54	0.0039

**Figure 3:**
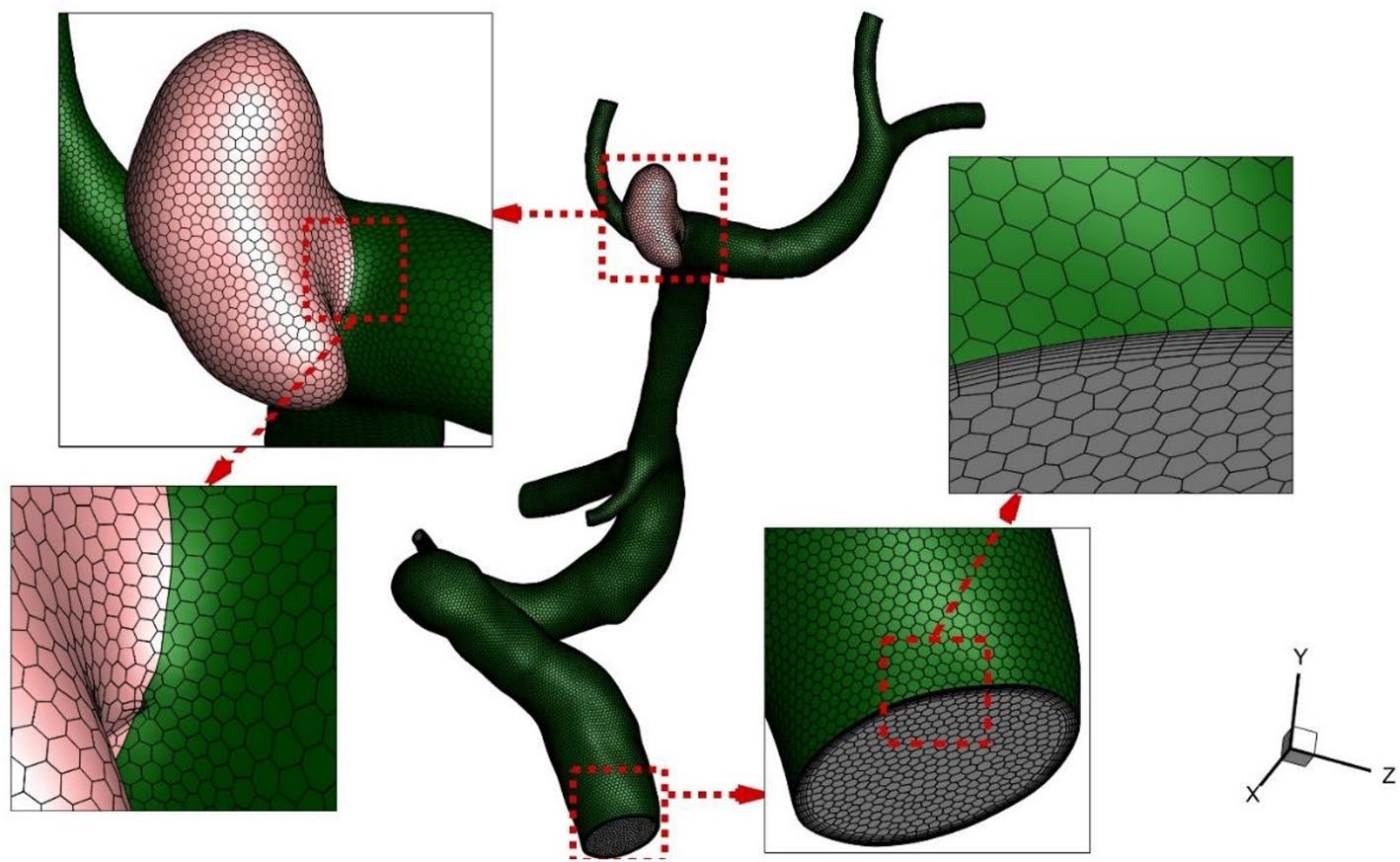
Grid production.

Figure 3 illustrated the produced grid for the selected cases with a close-up view. In a close-up view, the quality of produced grids is shown. In the produced grids, the size of the grid near the vessel wall is less than the center of the vessel since the main hemodynamic factors of WSS and OSI are calculated at the wall. In this figure, the sac section is separated for applying the coiling. A grid study is also done to investigate the mesh effects on our results. Table 2 presents the details of four produced grids and their effects on run time and average WSS on sac surfaces for two selected models. Grid analysis indicates that the model with 298201 cells and 402161 cells are appropriate grids for case A and case B, respectively.

**Table 2:** Grid study.

Case	Element size (mm)	Number of elements	Run-time (h)	Ave WSS on Sac (Pa)	Change %
A	0.24	85,476	2	6.04	-
	0.20	165,478	4	8.02	32.8
	0.16	298,201	6	9.13	13.8
	0.12	654,781	14	9.22	1.0
B	0.22	121,459	2	5.17	-
	0.18	226,987	4	7.34	42.0
	0.14	402,161	8	8.47	15.4
	0.10	805,794	18	8.51	0.5

The technique of endovascular coiling is implemented by filling the sac area with uniform porosity. Hence, the permeability of this domain is calculated via the capillary theory of kozeny^29,31^. Details of applied porosity for the selected MCA aneurysms are presented in Table 3. The present work investigated the impacts of two coiling porosities on the hemodynamic of the blood stream. The length and diameter of Coil are 30 cm and 0.254 mm, respectively.

**Table 3:** Porosity calculation- effect of coil.

Case	Aneurysm volume (mm^3^)	Porosity	Permeability (m^2^)	1/Permeability (1/m^2^)
A	25.5	0.5	7.09E−10	1.41E+09
		0.75	2.39E−09	4.17E+08
B	33.4	0.5	1.21E−09	8.21E+08
		0.75	4.10E−09	2.43E+08

**Figure 4:**
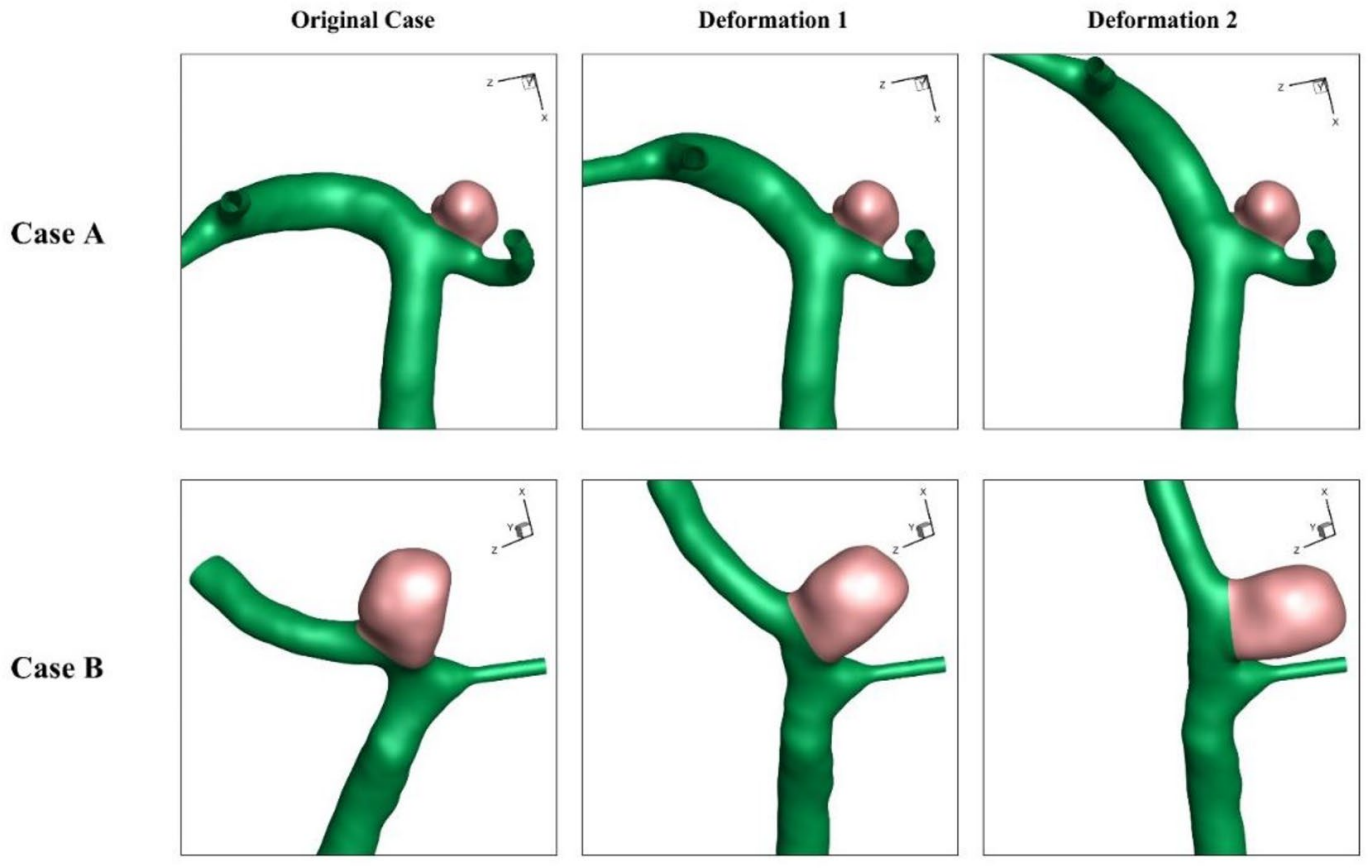
Deformed aneurysms.

The influence of the stent deformation is investigated in two different configurations. In fact, the angle of the parent vessel is changed in which the angle of the parent vessel orientation with the normal vector of the neck plane varies. In fact, the parent vessel becomes straight in the 2nd deformation as demonstrated in Fig. 4.

## Results and discussion

**Figure 5:**
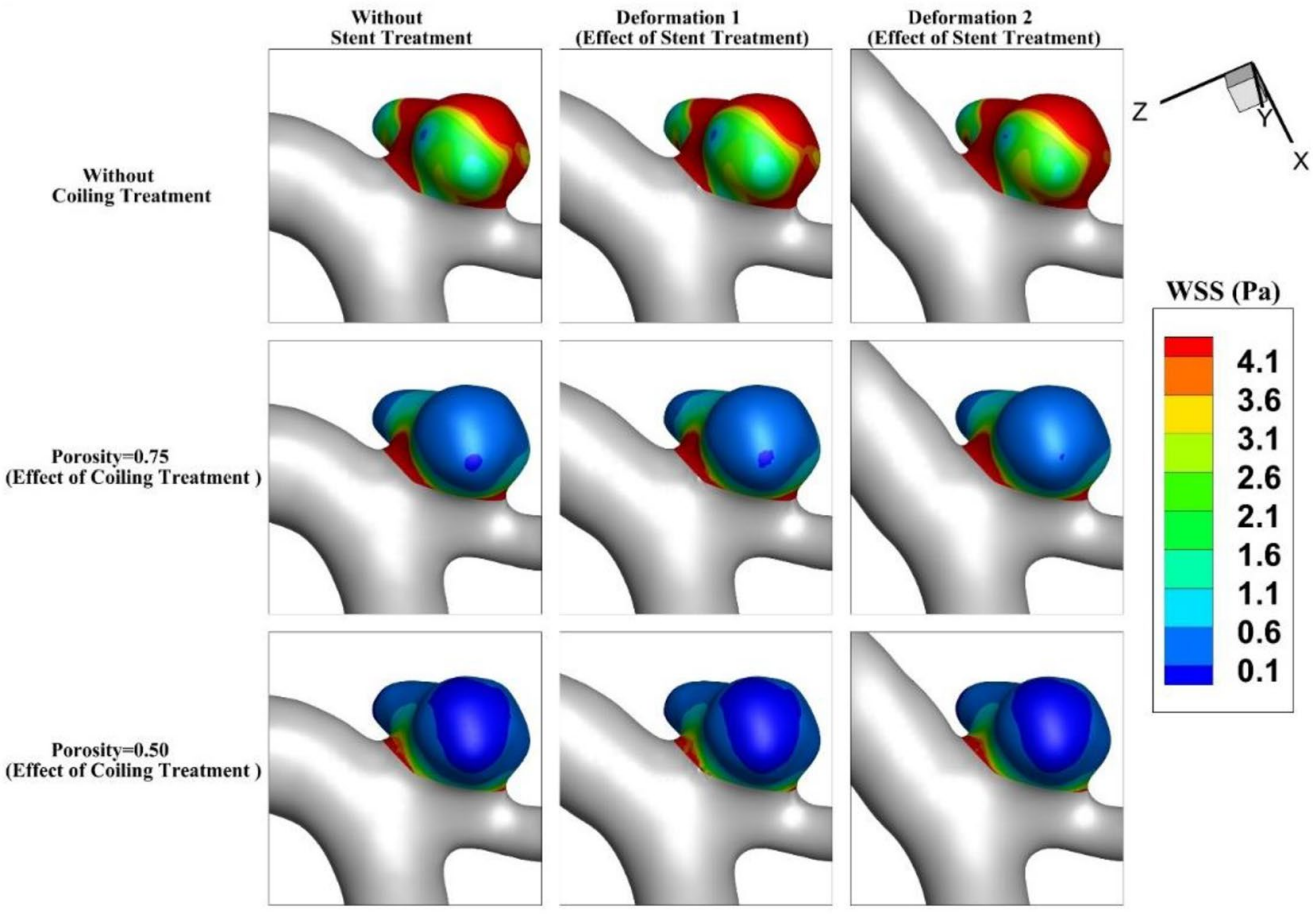
Wall shear stress (WSS) contour of Case A at Peak systolic (stage b).

**Figure 6:**
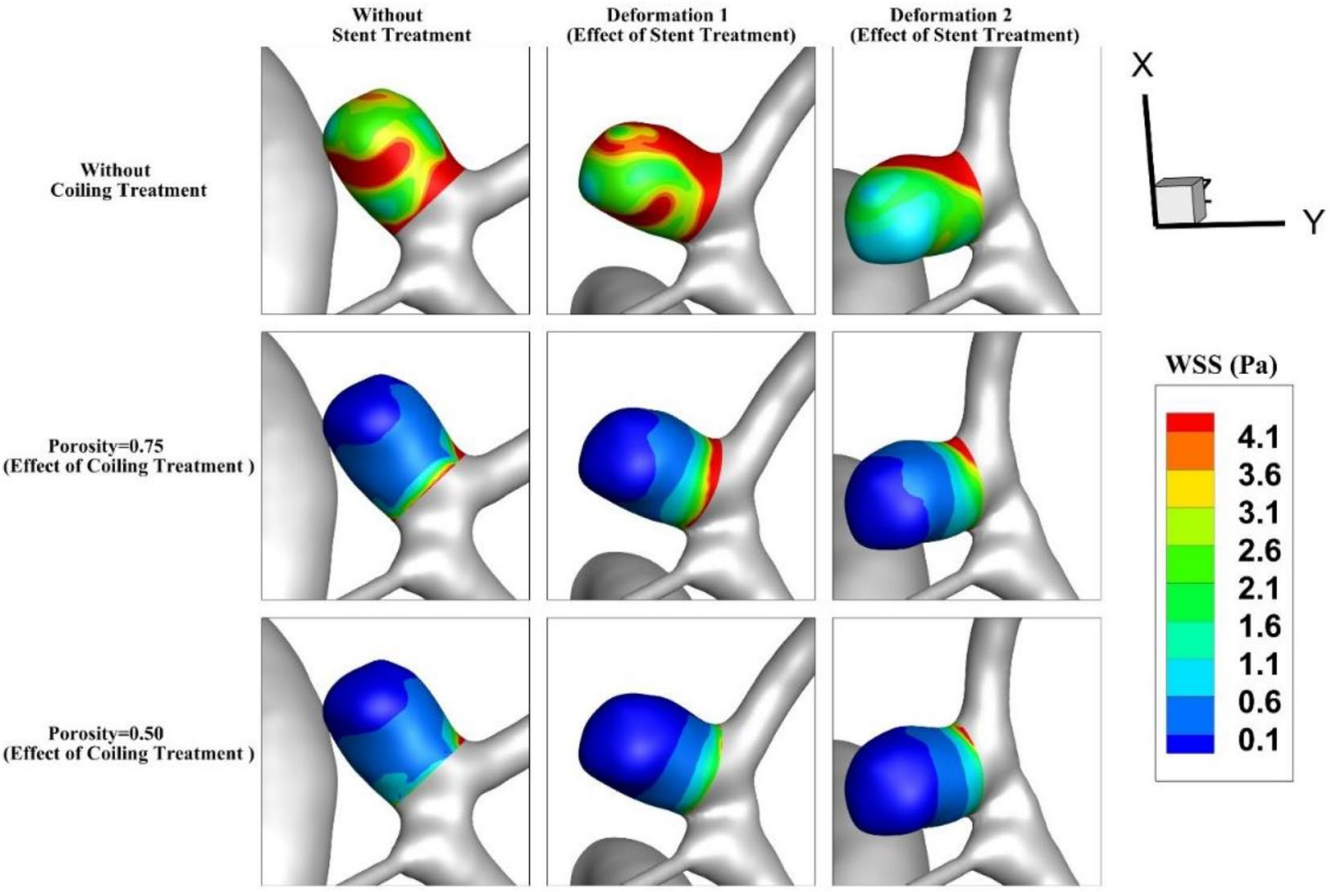
Wall shear stress (WSS) contour of Case B at Peak systolic (stage b).

Figure 5 illustrates the contour of the WSS on the sac wall for two coiling porosities of 0.75 and 0.5 for the selected aneurysm (case A) with/without stent deformation. The distribution of the WSS on the sac surface at peak systolic confirms that the influence of deformation on the distribution of WSS without fillings is not noticeable. As the coiling porosity is applied in the sac domain, the region with high WSS is restricted in the neck area where the flow rate of the blood is more than in other sections. In addition, the deformation impacts are more visible on models with lower porosity. In Fig. 6, the effects of aneurysm deformation on the WSS of the sac wall for case B are presented. The results indicate that deformation in this case has great impacts on the location and size of critical regions with high WSS on the sac surface. The impacts of the coiling porosity are substantial when the aneurysm deformation is done. The blood flow diverts efficiently in this case and this reduces the WSS on the sac surface.

**Figure 7:**
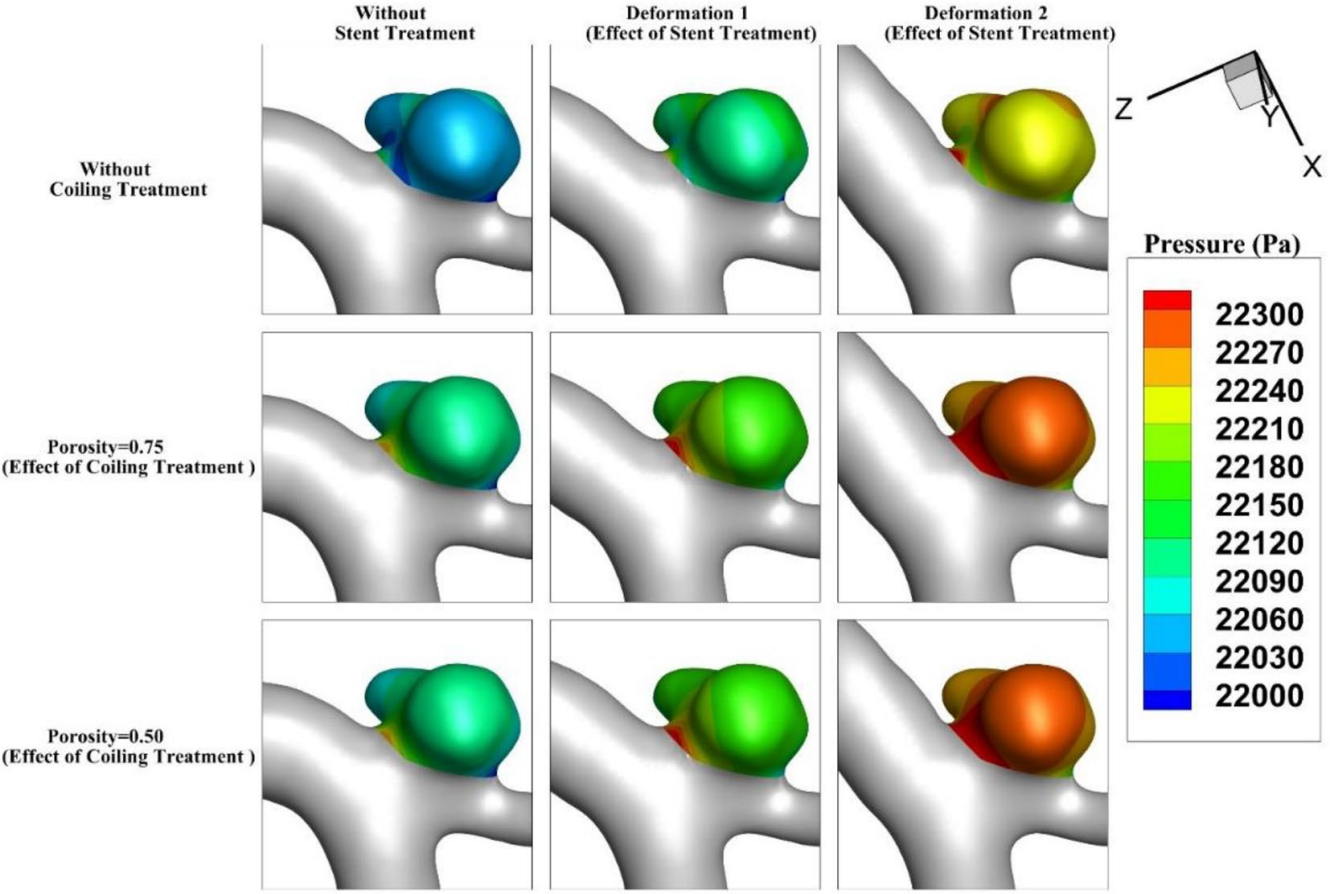
Pressure contour of Case A at Peak systolic (stage b).

**Figure 8:**
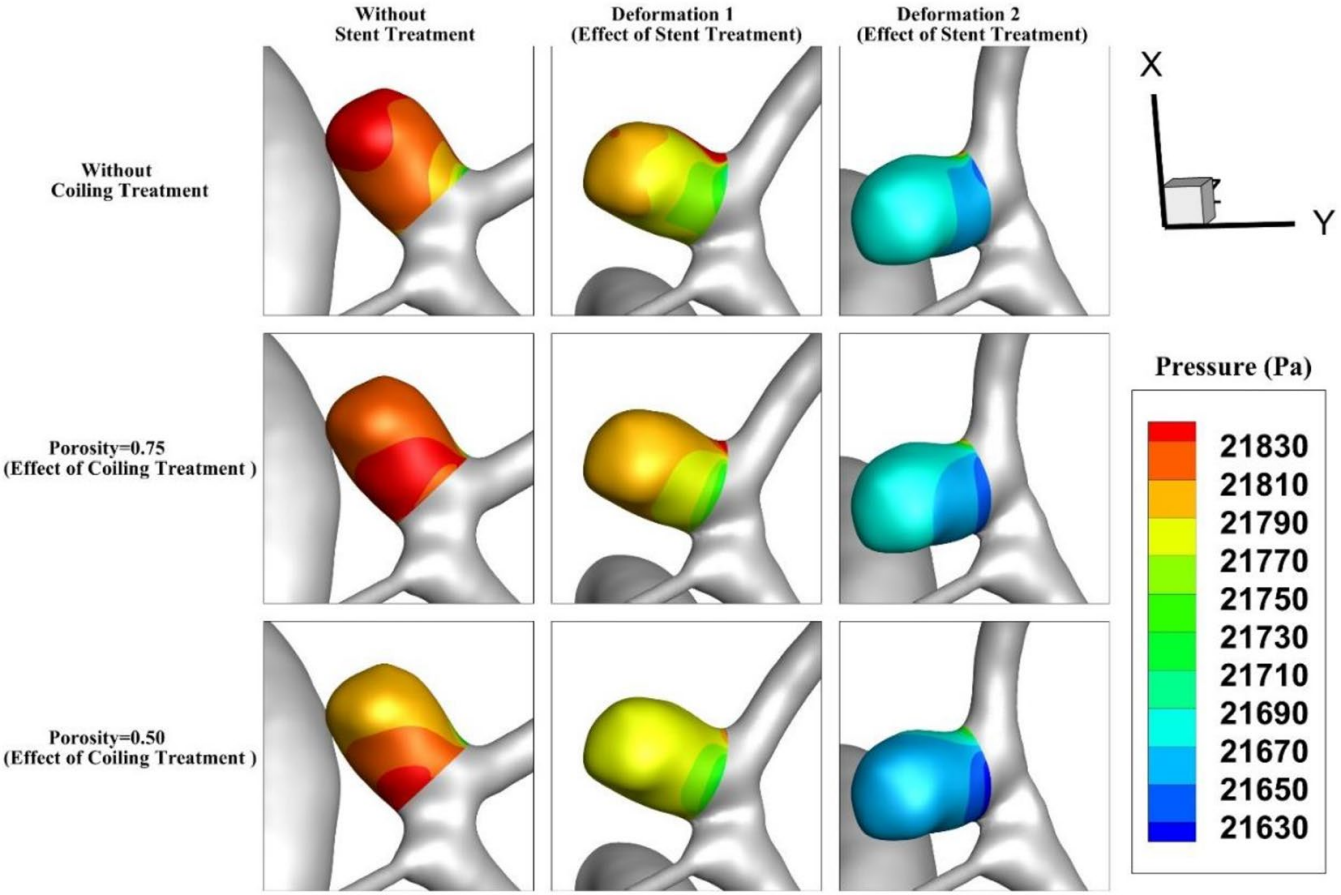
Pressure contour of Case B at Peak systolic (stage b).

The pressure contour for the aneurysm (case A) under the impacts of deformation and coiling is displayed in Fig. 7. The effects of aneurysm deformation of the pressure contour show that the deformations greatly reduce the pressure on the sac wall. Meanwhile, the porosity effects on the pressure distribution are not substantial as shown in the figure. In Fig. 8, the impacts of deformation and coiling on the pressure distribution of the MCA aneurysm (case B) are demonstrated. As mentioned before, the defamation of an aneurysm in this case efficiently occluded blood flow into the sac and this reduces the pressure value on the sac wall.

**Figure 9:**
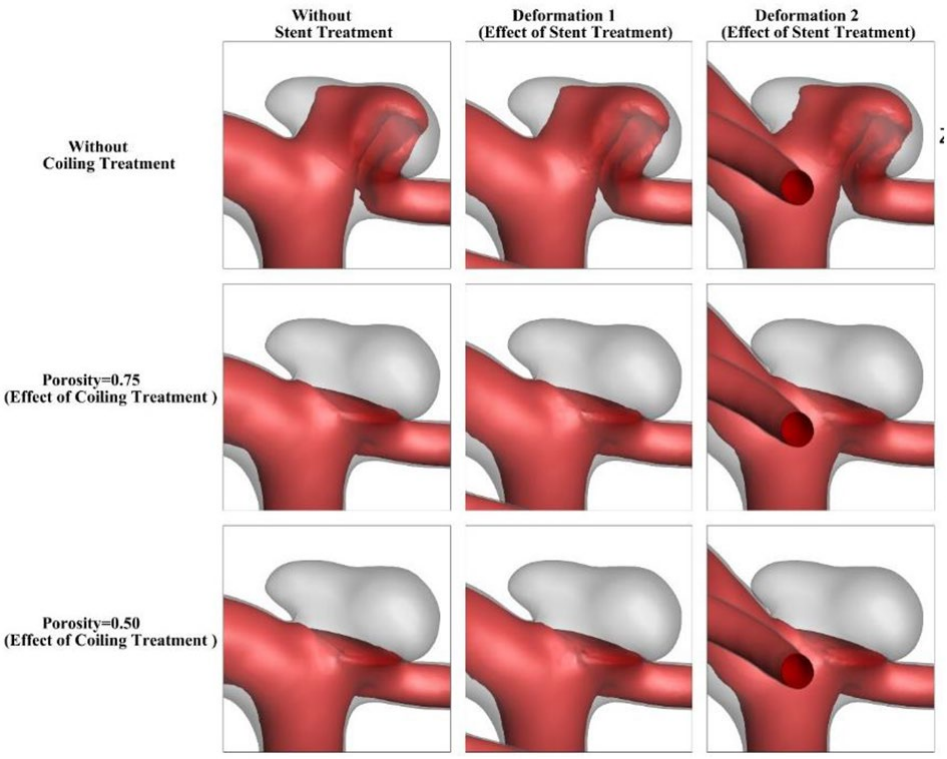
Iso-velocity (*V* = 0.2 m/s) blood contour of Case A at Peak systolic (stage b).

**Figure 10:**
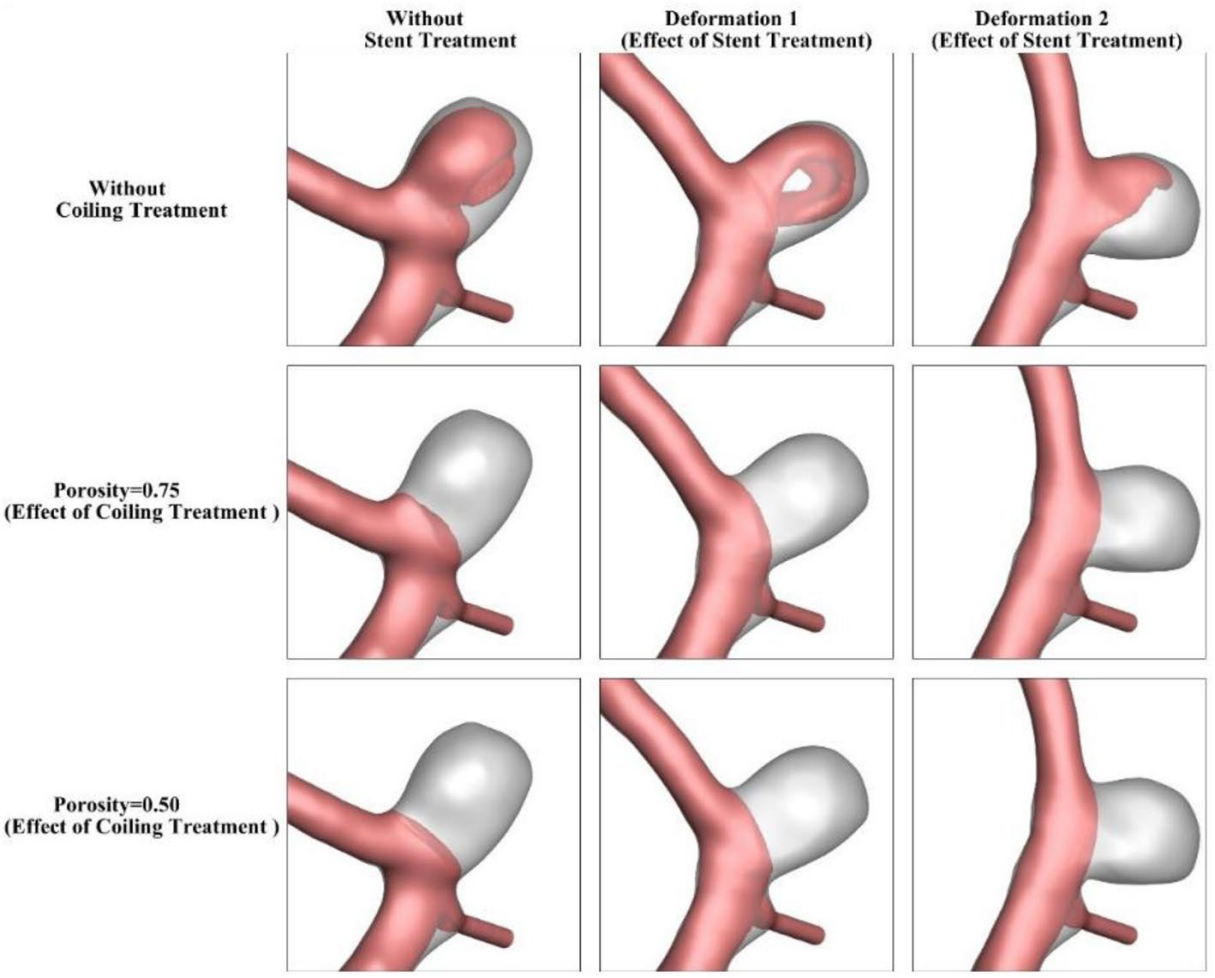
Iso-velocity (*V* = 0.15 m/s) blood contour of Case A at Peak systolic (stage b).

To understand the role of deformation on the hemodynamic of the MCA aneurysm, Fig. 9 displays the structure of the blood flow by iso-velocity surfaces in different porosities of coiling. In the original model (without deformation), deformation of the parent vessel does not change the blood feature. As the coiling porosity applied in the sac area, the velocity of blood decreases inside the domain and this reduces WSS on the sac wall. The blood feature of the case B under impacts of coiling and deformation shows how the flow diversion could protect the sac from rupture (Fig. 10). Since the blood entrance into the sac area is limited by deformation, the impacts of coiling porosity is not visible in different coiling porosities.

**Figure 11:**
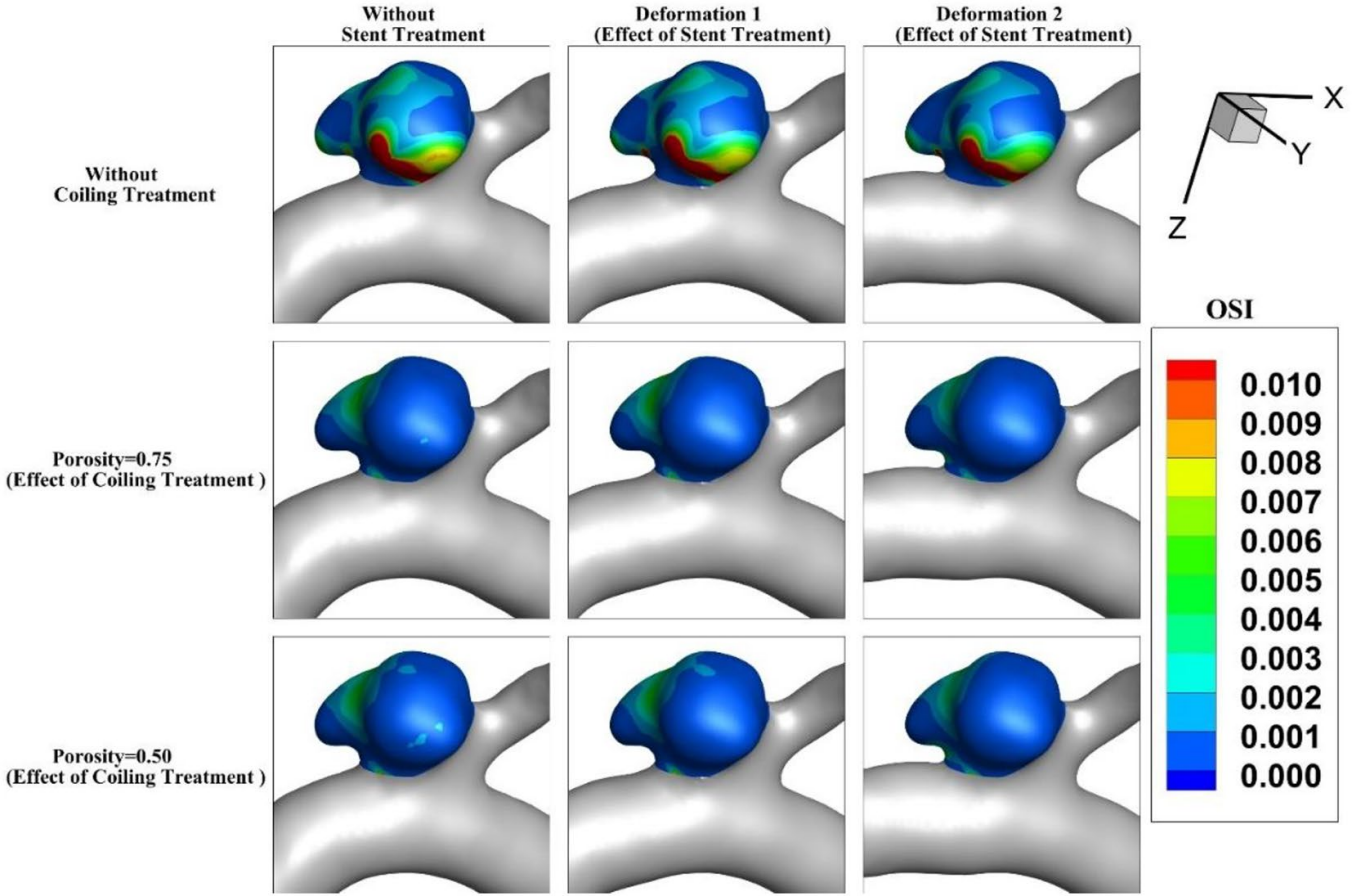
OSI contour of Case A at end of cardiac cycle (stage d).

**Figure 12:**
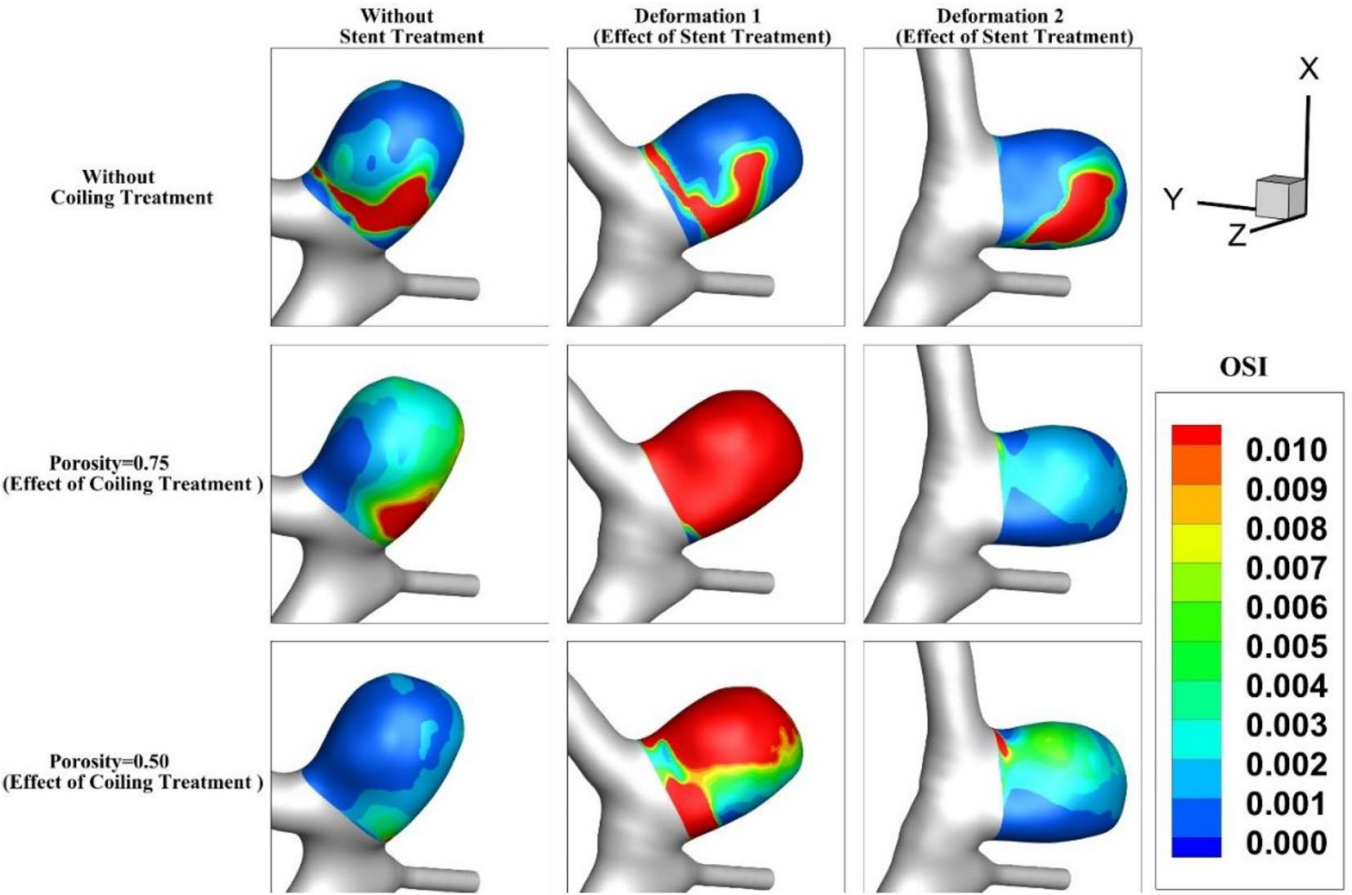
OSI contour of Case B at end of cardiac cycle (stage d).

OSI index is critical for the evaluation of the aneurysm rupture. Figure 11 demonstrates the contour of OSI at end of the cardiac cycle for an MCA aneurysm (Case A) when aneurysm deformation happens. In the original case (without coiling treatment), the value of OSI does not change meaningfully even after 2nd deformation and the critical region remains at the dome of the aneurysm. This pattern is preserved for cases with coil fillings. The blood entrance does not change due to the deformation of the parent vessel. Figure 12 illustrates the contour of the OSI at end of the cardiac cycle after deformations with/without coiling (Case B). It seems that the OSI index increases after 1st deformation while a substantial decrease is noticed after 2nd deformation.

**Figure 13:**
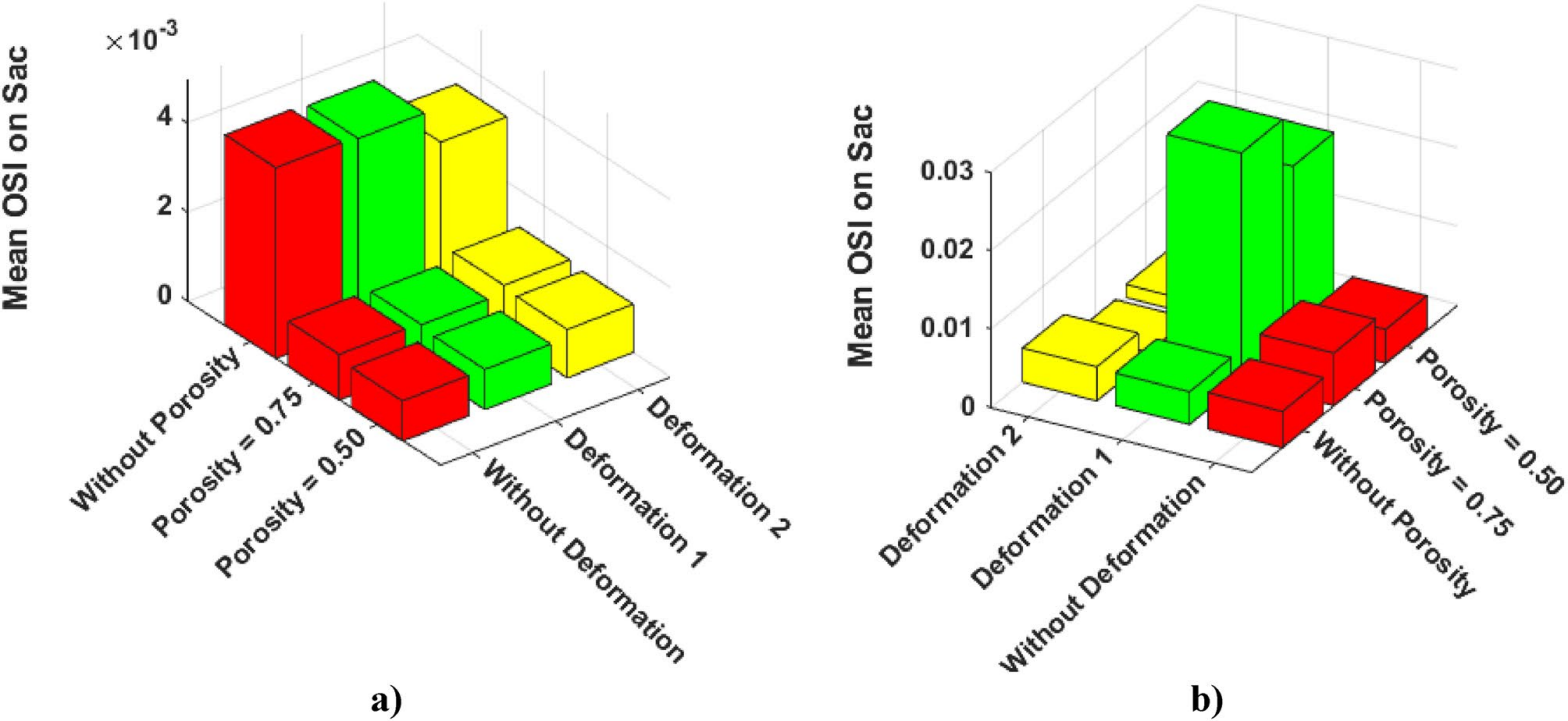
Effects of coiling porosity and stent-induced deformation on mean OSI value on aneurysm wall in (**a**) Case A, (**b**) Case B.

The results of mean OSI on sac wall for different coiling porosities under impacts of deformations are presented in Fig. 13. In both cases (A and B), the value of the OSI index substantially decreases by coiling rather than deformations. This is due to the role of coiling occlusion which reduces blood shear near the aneurysm wall. In the case of B, the effects of 1Ststage of deformation are not favorable as mentioned in the hemodynamic analysis.

**Figure 14:**
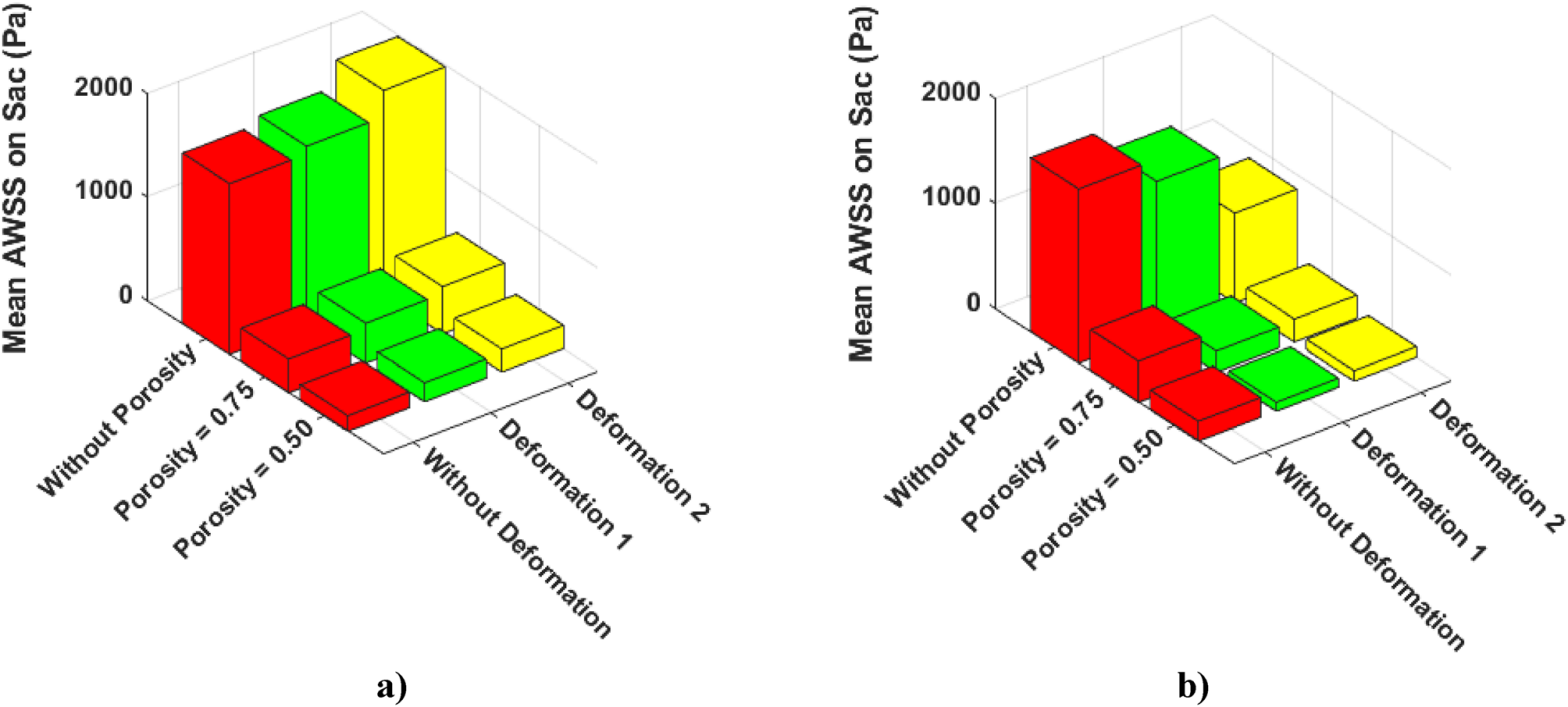
Effects of coiling porosity and stent-induced deformation on mean AWSS value on aneurysm wall in (**a**) Case A, (**b**) Case B.

In Fig. 14, a quantitative comparison of the mean AWSS on the sac surface of case A and B are demonstrated. In Case A, the mean value of AWSS slightly increases after deformations. However, the results of the mean AWSS on case B show that deformation of the parent vessel significantly decreases AWSS on the sac surface.

## Results and discussion

The impacts of stent-induced deformation on the hemodynamics of two distinctive MCA aneurysms are fully investigated in the present research. This study examined and explored the role of endovascular coiling when the parent vessel is deformed by the implementation of stents on the parent vessel near the sac. The modeling of the bloodstream in vessels and aneurysms is done via the computational fluid dynamic technique. The blood flow feature and WSS of aneurysm are compared to disclose the influence of coiling after post-interventional deformation. Presented results show that the effects of MCA aneurysm deformation are not always favourable for occlusion of the blood entrance. A comparison of coiling and deformations indicate that the coiling of an aneurysm would effectively reduce the risk of MCA aneurysm rupture.

## Data Availability

All data generated or analysed during this study are included in this published article.

## References

[1] Rostamian A, Fallah K, Rostamiyan Y, Alinejad J (2023). Computational study of the blood hemodynamic inside the cerebral double dome aneurysm filling with endovascular coiling. Sci. Rep..

[2] Shen X-Y, Xu H-Q, Gerdroodbary MB, Mousavi SV, Abazari AM, Imani SM (2022). Numerical simulation of blood flow effects on rupture of aneurysm in middle cerebral artery. Int. J. Modern Phys. C.

[3] Li C, Lin L, Zhang L, Xu R, Chen X, Ji J, Li Y (2021). Long noncoding RNA p21 enhances autophagy to alleviate endothelial progenitor cells damage and promote endothelial repair in hypertension through SESN2/AMPK/TSC2 pathway. Pharmacol. Res..

[4] Zeng Q, Bie B, Guo Q, Yuan Y, Han Q, Han X, Zhou X (2020). Hyperpolarized Xe NMR signal advancement by metal-organic framework entrapment in aqueous solution. Proc. Natl. Acad. Sci..

[5] Zhang X, Qu Y, Liu L, Qiao Y, Geng H, Lin Y, Zhao J (2021). Homocysteine inhibits pro-insulin receptor cleavage and causes insulin resistance via protein cysteine-homocysteinylation. Cell Rep..

[6] Yang B, Li Y, Zheng W, Yin Z, Liu M, Yin L, Yin C (2023). Motion prediction for beating heart surgery with GRU. Biomed. Signal Process. Control.

[7] Liu M, Zhang X, Yang B, Yin Z, Liu S, Yin L, Zheng W (2023). Three-dimensional modeling of heart soft tissue motion. Appl. Sci..

[8] Ban Y, Wang Y, Liu S, Yang B, Liu M, Yin L, Zheng W (2022). 2D/3D multimode medical image alignment based on spatial histograms. Appl. Sci..

[9] Hu F, Shi X, Wang H, Nan N, Wang K, Wei S, Zhao S (2021). Is health contagious?—based on empirical evidence from china family panel studies' data. Front. Public Health.

[10] Salavatidezfouli S, Alizadeh A, Gerdroodbary MB, Sabernaeemi A, Abazari AM, Sheidani A (2023). Investigation of the stent induced deformation on hemodynamic of internal carotid aneurysms by computational fluid dynamics. Sci. Rep..

[11] Wang Y, Zhai W, Zhang H, Cheng S, Li J (2023). Injectable polyzwitterionic lubricant for complete prevention of cardiac adhesion. Macromol. Biosci..

[12] Wang Y, Zhai W, Cheng S (2023). Surface-functionalized design of blood-contacting biomaterials for preventing coagulation and promoting hemostasis. Friction.

[13] Hariri S, Poueinak MM, Hassanvand A, Gerdroodbary MB, Faraji M (2023). "Effects of blood hematocrit on performance of endovascular coiling for treatment of middle cerebral artery (MCA) aneurysms: Computational study. Interdiscipl. Neurosurg..

[14] Valipour P (2022). Effects of coiling embolism on blood hemodynamic of the MCA aneurysm: A numerical study. Sci. Rep..

[15] Rostamian A, Fallah K, Rostamiyan Y (2023). Reduction of rupture risk in ICA aneurysms by endovascular techniques of coiling and stent: Numerical study. Sci. Rep..

[16] Sabernaeemi A, Gerdroodbary MB, Salavatidezfouli S, Valipour P (2023). Influence of stent-induced vessel deformation on hemodynamic feature of bloodstream inside ICA aneurysms. Biomech. Model. Mechanobiol..

[17] Voss S, Oliver B, Gábor J, Philipp B (2019). Stent-induced vessel deformation after intracranial aneurysm treatment—a hemodynamic pilot study. Comput. Biol. Med..

[18] Wang HS (2021). The effect of drug position on the properties of paclitaxel-conjugated gold nanoparticles for liver tumor treatment. Chin. Chem. Lett..

[19] Fallah K, Fardad A, Fattahi E, Zadeh NS, Ghaderi A (2012). Numerical simulation of planar shear flow passing a rotating cylinder at low Reynolds numbers. Acta Mech..

[20] Allahyari S, Ghaderi A, Behzadmehr A, Fallah K, Nasimi MH, Hassani M (2023). Investigating the effects of nanoparticles mean diameter on laminar mixed convection of a nanofluid through an inclined tube with circumferentially nonuniform heat flux. J. Eng. Thermophys..

[21] Sadeh A, Kazemi A, Khoo MB, Gerdroodbary MB (2023). Computational analysis of the blood hemodynamic inside internal cerebral aneurysm in the existence of endovascular coiling. Int. J. Modern Phys. C.

[22] Yu Y, Wang L, Ni S, Li D, Liu J, Chu HY, Zhang G (2022). Targeting loop3 of sclerostin preserves its cardiovascular protective action and promotes bone formation. Nature Commun..

[23] Wang L, Yu Y, Ni S, Li D, Liu J, Xie D, Chu HY, Ren Q, Zhong C, Zhang N, Li N, Sun M, Zhang ZK, Zhuo Z, Zhang H, Zhang S, Li M, Xia W, Zhang Z, Chen L, Zhang G (2022). Therapeutic aptamer targeting sclerostin loop3 for promoting bone formation without increasing cardiovascular risk in osteogenesis imperfecta mice. Theranostics.

[24] Gao Z, Pan X, Shao J, Jiang X, Su Z, Jin K, Ye J (2022). Automatic interpretation and clinical evaluation for fundus fluorescein angiography images of diabetic retinopathy patients by deep learning. Br. J. Ophthalmol..

[25] Ao J, Shao X, Liu Z, Liu Q, Xia J, Shi Y, Ji M (2023). Stimulated Raman scattering microscopy enables gleason scoring of prostate core needle biopsy by a convolutional neural network. Cancer Res..

[26] Xu H, Van der Jeught K, Zhou Z, Zhang L, Yu T, Sun Y, Lu X (2021). Atractylenolide I enhances responsiveness to immune checkpoint blockade therapy by activating tumor antigen presentation. J. Clin. Investig..

[27] Hu J (2021). The progress and perspective of strategies to improve tumor penetration of nanomedicines. Chin. Chem. Lett..

[28] AneuriskWeb project website. http://ecm2.mathcs.emory.edu/aneuriskweb. Emory University, Department of Math&CS (2012).

[29] Malvè M, Chandra S, García A, Mena A, Martínez MA, Finol EA, Doblaré M (2014). Impedance-based outflow boundary conditions for human carotid haemodynamics. Comput. Methods Biomech. Biomed. Eng..

[30] Mitsos AP, Nikolaos MPK, Ventikos YP, Byrne JV (2008). Haemodynamic simulation of aneurysm coiling in an anatomically accurate computational fluid dynamics model. Neuroradiology.

[31] Boccadifuoco A, Mariotti A, Celi S, Martini N, Salvetti MV (2018). Impact of uncertainties in outflow boundary conditions on the predictions of hemodynamic simulations of ascending thoracic aortic aneurysms. Comput. Fluids.

[32] Shen X-Y, Gerdroodbary MB, Abazari AM, Moradi R (2021). Computational study of blood flow characteristics on formation of the aneurysm in internal carotid artery. Eur. Phys. J. Plus.

[33] Sadeh A, Kazemi A, Bahramkhoo M (2023). Computational study of blood flow inside MCA aneurysm with/without endovascular coiling. Sci. Rep..

[34] Rostamian A, Fallah K, Rostamiyan Y, Alinejad J (2022). Application of computational fluid dynamics for detection of high risk region in middle cerebral artery (MCA) aneurysm. Int. J. Modern Phys. C.

[35] Poueinak MM, Abdollahi SA, Alizadeh A, Youshanlui MA, Zekri H, Gerdroodbary MB (2023). Computational study of blood hemodynamic in ICA aneurysm with coiling embolism. Int. J. Mod. Phys. C.

[36] Xiao-Yong S, Gerdroodbary MB, Poozesh A, Abazari AM, Imani SM (2021). Effects of blood flow characteristics on rupture of cerebral aneurysm: Computational study. Int. J. Mod. Phys. C.

[37] Sheidani A, Gerdroodbary MB, Poozesh A, Sabernaeemi A, Salavatidezfouli S, Hajisharifi A (2022). Influence of the coiling porosity on the risk reduction of the cerebral aneurysm rupture: Computational study. Sci. Rep..

[38] Jin Z-H, Gerdroodbary MB, Valipour P, Faraji M, Abu-Hamdeh NH (2023). CFD investigations of the blood hemodynamic inside internal cerebral aneurysm (ICA) in the existence of coiling embolism. Alex. Eng. J..

[39] Khamseh AG, Sohrab AG (2018). Experimental and modeling investigation of thorium biosorption by orange peel in a continuous fixed-bed column. J. Radioanal. Nucl. Chem..

[40] Fallah K, Fattahi E (2022). Splitting of droplet with different sizes inside a symmetric T-junction microchannel using an electric field. Sci. Rep..

[41] Qiu SH (2022). Single-cell level point mutation analysis of circulating tumor cells through droplet microfluidics. Chin. Chem. Lett..

[42] Sadeghi A, Amini Y, Saidi MH, Chakraborty S (2014). Numerical modeling of surface reaction kinetics in electrokinetically actuated microfluidic devices. Anal. Chim. Acta.

[43] Jin RH (2021). Redox-responsive micelles integrating catalytic nanomedicine and selective chemotherapy for effective tumor treatment. Chin. Chem. Lett..

[44] Jiang H, Zhiwei L, Gerdroodbary MB, Sabernaeemi A, Salavatidezfouli S (2023). The influence of sac centreline on saccular aneurysm rupture: Computational study. Sci. Rep..

[45] Liu M, Li C, Zhang Y, Yang M, Gao T, Cui X, Wang X, Xu W, Zhou Z, Liu B, Said Z, Li R, Sharma S (2022). Analysis of grinding mechanics and improved grinding force model based on randomized grain geometric characteristics. Chin. J. Aeronaut..

[46] Sadeghi A, Amini Y, Saidi MH, Yavari H (2015). Shear-rate-dependent rheology effects on mass transport and surface reactions in biomicrofluidic devices. AIChE J..

